# Development and Evaluation of a Genome-Wide 6K SNP Array for Diploid Sweet Cherry and Tetraploid Sour Cherry

**DOI:** 10.1371/journal.pone.0048305

**Published:** 2012-12-20

**Authors:** Cameron Peace, Nahla Bassil, Dorrie Main, Stephen Ficklin, Umesh R. Rosyara, Travis Stegmeir, Audrey Sebolt, Barbara Gilmore, Cindy Lawley, Todd C. Mockler, Douglas W. Bryant, Larry Wilhelm, Amy Iezzoni

**Affiliations:** 1 Department of Horticulture, Washington State University, Pullman, Washington, United States of America; 2 USDA-ARS, National Clonal Germplasm Repository, Corvallis, Oregon, United States of America; 3 Department of Horticulture, Michigan State University, East Lansing, Michigan, United States of America; 4 Illumina Inc., Hayward, California, United States of America; 5 The Donald Danforth Plant Science Center, St. Louis, Missouri, United States of America; 6 Intuitive Genomics, Inc., St. Louis, Missouri, United States of America; 7 Oregon Health Sciences University, Portland, Oregon, United States of America; Pennsylvania State University, United States of America

## Abstract

High-throughput genome scans are important tools for genetic studies and breeding applications. Here, a 6K SNP array for use with the Illumina Infinium® system was developed for diploid sweet cherry (*Prunus avium*) and allotetraploid sour cherry (*P. cerasus*). This effort was led by RosBREED, a community initiative to enable marker-assisted breeding for rosaceous crops. Next-generation sequencing in diverse breeding germplasm provided 25 billion basepairs (Gb) of cherry DNA sequence from which were identified genome-wide SNPs for sweet cherry and for the two sour cherry subgenomes derived from sweet cherry (*avium* subgenome) and *P. fruticosa* (*fruticosa* subgenome). Anchoring to the peach genome sequence, recently released by the International Peach Genome Initiative, predicted relative physical locations of the 1.9 million putative SNPs detected, preliminarily filtered to 368,943 SNPs. Further filtering was guided by results of a 144-SNP subset examined with the Illumina GoldenGate® assay on 160 accessions. A 6K Infinium® II array was designed with SNPs evenly spaced genetically across the sweet and sour cherry genomes. SNPs were developed for each sour cherry subgenome by using minor allele frequency in the sour cherry detection panel to enrich for subgenome-specific SNPs followed by targeting to either subgenome according to alleles observed in sweet cherry. The array was evaluated using panels of sweet (n = 269) and sour (n = 330) cherry breeding germplasm. Approximately one third of array SNPs were informative for each crop. A total of 1825 polymorphic SNPs were verified in sweet cherry, 13% of these originally developed for sour cherry. Allele dosage was resolved for 2058 polymorphic SNPs in sour cherry, one third of these being originally developed for sweet cherry. This publicly available genomics resource represents a significant advance in cherry genome-scanning capability that will accelerate marker-locus-trait association discovery, genome structure investigation, and genetic diversity assessment in this diploid-tetraploid crop group.

## Introduction

Within *Prunus* (Rosaceae), two cherry species, sweet (*P. avium*) and sour cherry (*P. cerasus*), are highly valued for their excellent quality fruit. These two species represent a natural diploid-tetraploid series with the tetraploid sour cherry (2n = 4*x* = 32) arising through natural hybridization between sweet cherry (2n = 2*x* = 16) and the wild tetraploid ground cherry (*P. fruticosa*) [1], [2]. In cherry, linkage maps constructed for the genetically less complex sweet cherry are primarily based on simple sequence repeat (SSR) markers [3], [4], [5], [6]. The application of these linkage maps for other studies such as quantitative trait locus (QTL) discovery is limited by the low-throughput and, in many cases, low density and low levels of polymorphism in cultivated sweet cherry germplasm for the SSR markers.

High-throughput and low-cost next generation sequencing (NGS) is a powerful approach to identify single nucleotide polymorphisms (SNPs) which can be used as markers for the development of high-density genome scans. This approach has been successfully used to develop genome scan platforms for diploid crops such as apple [7], maize [8], rice [9] and polyploid crops such as potato [10], [11], and wheat [12]. While low diversity in a crop still reduces the proportion of observed polymorphic markers, the sheer number of markers that can be efficiently generated by the NGS approach overcomes this historical limitation. A 9K publicly available SNP array for peach, recently developed by an international consortium, is widely being used for advanced genetic studies of peach [13]. The development of this array was facilitated by release of the peach (*Prunus persica*) reference genome sequence by the International Peach Genome Initiative [14].The high quality of the peach sequence was reinforced by high congruence between genetic and physical map positions of peach array SNPs (I. Verde, pers. comm.).

Extensive synteny conserved among the diploid *Prunus* species [3] suggests that the peach genome sequence can be used as a template to develop a genome-wide set of markers for *Prunus* crops for which a high quality genome sequence is not available. For example, comparisons of available sweet cherry genetic maps with the high-density peach×almond “T×E” *Prunus* reference genetic map [3]identified extensive co-linearity [4], yet complete co-linearity could not be rigorously tested as the largest sweet cherry population used consisted of just 118 individuals [6]. Dirlewanger et al. [15] have estimated the genome size of sweet cherry to be 338 Mb.

Herein we use a cherry-peach comparative genomics strategy to develop a moderate-density cherry SNP array relevant for sweet and sour cherry breeding germplasm based on SNPs discovered using next generation sequencing platforms. This effort was led by RosBREED, a community initiative to enable marker-assisted breeding for rosaceous crops [16] which led the recent equivalent developments of SNP arrays for apple [7] and peach [13].

## Materials and Methods

**Figure 1:**
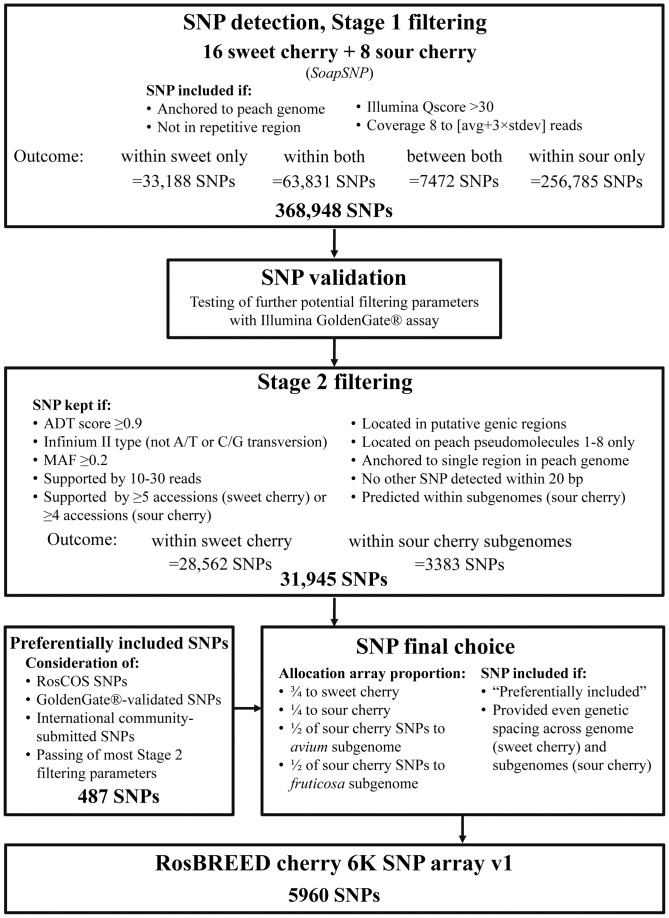
Workflow for SNP detection, validation, and final choice in development of the RosBREED 6K cherry SNP array v1. Stage 1 filtered 1.9 million cherry SNPs anchored to the peach genome to almost 40K SNPs. More stringent filtering criteria in Stage 2, guided by a prior validation step with a small SNP subset examined for a range of potential filters, putatively enriched the quality of the remaining 32K SNP pool. Finally, the 6K array SNPs were chosen from among stage 2 SNPs by attempting to achieve even genetic spacing over species genomes and subgenomes with pre-determined proportional allocations, after preferential inclusion of certain SNPs. ADT = Illumina's Assay Design Tool. MAF = minor allele frequency.

The workflow and design parameters described below are summarized in Figure 1.

### Whole genome re-sequencing of cherry breeding accessions

A SNP detection panel of 16 sweet and 8 sour cherry accessions was chosen for whole genome, low-coverage resequencing (Table 1). The accessions were founders, intermediate ancestors, or important parents used in U.S breeding programs. For each accession, paired-end libraries were prepared as recommended by manufacturer protocols (Illumina Inc., San Diego, CA, USA). For sweet cherry, equimolar amounts of four libraries were pooled (to create four pools in total) while for sour cherry, with twice the genome size of sweet cherry, equimolar volumes of two libraries were pooled (to create another four pools in total) (Table 1). Each library pool was sequenced in one lane of Illumina GA II with 80 cycles per read at the Center for Genome Research and Biocomputing (CGRB; Oregon State University, Corvallis, OR, USA). The raw sequence data was retrieved and kept separate for each cherry accession and then aligned to the reference genome of ‘Lovell’ peach [14] using *SOAP* [17] with parameters of M = 4 (find best hits for each seed, ≤2 mismatches allowed), r = 1 (repeats aligned 1 time randomly), and v = 2 (≤2 mismatches allowed).

**Table 1:** Cherry accessions used for low-coverage re-sequencing and subsequent SNP detection.

Cherry type	Accession	Adaptors	Read count (million)	No. aligned reads (million)
Sweet	PMR-1	GGGT	10.9	1.5
Sweet	New York 54	TCGT	10.7	1.5
Sweet	Ambrunes	CCAT	8.8	1.2
Sweet	Schmidt	AGCT	11.3	1.6
Sweet	Regina	GGGT	10.1	1.3
Sweet	Van	TCGT	9.9	1.4
Sweet	Lambert	CCAT	9.0	1.3
Sweet	Gil Peck	AGCT	10.3	1.3
Sweet	Emperor Francis	GGGT	16.5	2.0
Sweet	Attika	TCGT	16.0	2.0
Sweet	Napoleon	CCAT	9.0	1.2
Sweet	Windsor	AGCT	20.8	2.7
Sweet	Cashmere	GTGT	16.5	2.4
Sweet	Krupnoplodnaya	TCGT	13.7	1.8
Sweet	Black Republican	AGCT	1.6	0.2
Sweet	Early Burlat	CCAT	20.5	2.9
Sour	Újfehértói Fürtös	GGGT	5.8	0.7
Sour	Montmorency	TCCT	5.7	0.8
Sour	Rheinische Schattenmorelle	CCAT	16.0	2.2
Sour	23 23 (13)	AGCT	5.1	0.8
Sour	R1 (1)	GTGT	26.1	3.9
Sour	Nana	AACT	22.9	3.6
Sour	Englaise Timpurii	TGGT	13.7	2.4
Sour	Pitic de Iasi	CCCT	17.6	2.8
Total			308.6	43.5

The adaptors (used to retrieve accession-specific sequences from pools), numbers of Illumina Genome Analyzer II reads, and numbers of reads aligned to the peach ‘Lovell’ genome are indicated.

### Detection and Stage 1 filtering of SNPs

SNPs from resequenced accessions were detected using *SOAPsnp* (http://soap.genomics.org.cn/soapsnp.html) as recommended by Li et al. [17] The detected SNPs were filtered (“Stage 1” filtering) and kept if: (1) *SOAPsnp*'s “quality score” metric of the consensus genotype was greater than 30; (2) the sequencing depth at the putative SNP position was at least 8; and (3) the sequencing depth at the putative SNP position was no greater than 1254, corresponding to the average read depth of all SNPs plus three standard deviations. For each gene, the exonic, intronic, and intergenic space was defined using the Peach v1.0 ‘dhLovell’ genome annotation [14]. This filtration yielded “Stage 1 SNPs”.

### SNP validation with the GoldenGate® assay

A set of 144 SNPs were chosen from among Stage 1 SNPs to validate the efficiency of SNP detection and adjust subsequent filtering parameters (Figure 1). The initial choice comprised 80 Stage 1 SNPs evenly spaced over the sweet cherry genome and 40 SNPs evenly spaced over the sour cherry genome. In the absence of the availability of a whole genome sequence for cherry, the whole genome sequence of peach, i.e., the eight pseudomolecules of the haploid chromosomes and linkage groups (LGs) of the Peach v1.0 ‘dhLovell’ genome assembly [14], was used as the proxy cherry genome. One sweet cherry SNP was chosen to be located within 100 thousand basepairs (kb) from each end of each LG. An even number of sweet cherry SNPs chosen between these ends were then evenly physically spaced along each LG according to LG genetic distances of [3], corresponding to one SNP every 2–4 million basepairs (Mb). The spacing of the 80 sweet cherry SNPs across all LGs averaged 3.00 Mb (standard deviation of 0.52 Mb), with a minimum average of 2.40 Mb (±0.11 Mb) for LG8 and a maximum average of 3.8 Mb (±0.09 Mb) for LG2. A total of 40 sour cherry SNPs (many of which were also SNPs for sweet cherry) were chosen equidistant between pairs of sweet cherry SNPs (first and second, third and fourth, etc.) such that the average physical distance between sour cherry SNPs was twice that of sweet cherry. For both crop sources of the 120 SNPs, 40% were chosen to be located in exons (CDS) of annotated genes, 20% in introns, 20% in 5′ or 3′ untranslated regions (UTR, outside genes but within 2 kb of start or stop codons), and the final 20% in intergenic regions. Sixteen further sweet cherry SNPs and eight further sour cherry SNPs spanned an 863 kb region on LG2 between the simple sequence repeat markers CPSCT038 and BPPCT034, the location of a major trait locus associated with fruit size [18]. While these further 24 trait locus-targeted SNPs were chosen for variation in genic regions where possible, preference was given to achieving uniform physical spacing in the designated windows. Approximately 20% of the 144 validation SNPs were planned to be accession-specific, i.e., their minor allele would be detected in only one re-sequenced accession of the detection panel. Twenty-two of the sweet cherry SNPs and nine of the sour cherry SNPs met this criterion within accessions of their respective crops, and a further eight sweet cherry SNPs were accession-specific within sour cherry accessions of the detection panel. Accession-specific SNPs were from 12 of the 16 sweet cherry accessions and five of the eight sour cherry accessions. The 144 SNPs also deliberately included a wide range of minor allele frequencies (MAFs).

To examine filtering parameters for associations with genotyping efficiency, validation panels of 79 sweet cherry (Table S1) and 81 sour cherry (Table S2) accessions were genotyped for the 144 SNPs described above. Individuals in the validation panel were founders, intermediate ancestors, and important breeding parents of modern cherry cultivars and included the 24 accessions of the SNP detection panel. Genomic DNA was purified from each accession using the E-Z 96 Tissue DNA Kit (Omega Bio-Tek, Inc., Norcross, GA, USA). DNA was quantitated with the Quant-iT™ PicoGreen® Assay (Invitrogen, Carlsbad, CA, USA), using the Victor multiplate reader (Perkin Elmer Inc., San Jose, CA, USA). Concentrations were adjusted to a minimum of 50 ng/µl in 5 µl aliquots and were submitted to the Research Technology Support Facility at Michigan State University (East Lansing, MI, USA) where the GoldenGate® assay was performed following the manufacturer's protocol (Illumina Inc.). After amplification, PCR products were hybridized to VeraCode microbeads via the address sequence for detection on a BeadXpress Reader. SNP genotypes were scored with the Genotyping Module of GenomeStudio Data Analysis software v2010.3 [19].

### SNP final choice for RosBREED 6K array

Two further rounds of filtering were used, considering validation results. Stage 1 SNPs were converted to Illumina Assay Design Tool (ADT) format with custom scripts and their ADT scores calculated; only SNPs with a score ≥0.90 were retained. A/T and C/G transversions were removed from further consideration, thus retaining only SNPs of normalization bin “C” (for Infinium® II compatibility). SNPs with MAF <0.2 were discarded, as well as those not supported by between 10 and 30 reads and at least five detection panel accessions for sweet cherry or four accessions for sour cherry. Non-intragenic SNPs were removed for sweet cherry, i.e., only exonic and intronic SNPs were considered for the array. Non-intragenic SNPs were included in the final array for sour cherry, rather than intragenic, due to a mistake in filtering. SNPs not assigned to the first eight pseudomolecules representing peach's eight chromosomes were excluded. Non-uniquely anchored SNPs (those SNPs for which at least 90% of the 60 bp flanking sequences were anchored to more than one peach genome location) were also removed, and there could be no other detected SNP within 20 bp of the targeted SNP. For sour cherry, SNPs predicted to be between the *avium* and *fruticosa* subgenomes (i.e., AABB) or polymorphic in both subgenomes (i.e., ABAB) were removed (strategy described below). This filtering process yielded “Stage 2 SNPs” (Figure 1).

Subgenome specificity of sour cherry SNPs was deduced by the following strategy. MAF within the sour cherry detection panel was used as a proxy for allele dosage. SNPs close to a 1∶1 ratio for two alternative SNP alleles were assumed to represent either SNPs between the *avium* and *fruticosa* subgenomes (i.e., AABB), and therefore not expected to segregate, or SNPs segregating in both subgenomes (i.e., ABAB), as neither were desirable for the final array. Sour cherry SNPs were examined for divergence from a 1∶1 ratio by Chi-squared analysis, with those significantly different (p<0.05) assumed to represent subgenome-specific SNPs (i.e., ABAA or AAAB). Finally, to determine whether such SNPs were polymorphic within the *avium* or *fruticosa* subgenome, sequence information from sweet cherry detection panel accessions was consulted: presence of the rare sour cherry allele in sweet cherry was assumed to indicate that the SNP identified polymorphism within the *avium* subgenome; absence of the allele in sweet cherry indicated a *fruticosa* subgenome SNP.

Members of the international cherry genomics community requested inclusion of 487 SNPs in the final array. These “preferentially included SNPs” were: 304 sweet cherry RosCOS SNPs [6]; 150 Stage 1 SNPs spanning 3 Mb at a LG2 fruit size locus [18] and passing Stage 2 filtering criteria except for inclusion of: intergenic SNPs, any MAF, and sour cherry SNPs significantly different from 1∶1 dosage at p<0.10; 21 of the GoldenGate®-validated SNPs with high MAF; and 12 pre-validated SNPs from other research programs.

Choosing SNPs for the 6K SNP array considered the final number designated to each crop (approximately 75% to sweet cherry and 25% to sour cherry), their estimated genetic location, the subgenome targeted for sour cherry, and the two sources of available SNPs (i.e., Stage 2 SNPs and preferentially included SNPs). Preferentially included SNPs were automatically included, leaving 5513 SNPs to be chosen. For the ∼75% SNPs allocated to sweet cherry and ∼25% to sour cherry, SNPs were evenly spaced genetically across each crop's genome. For sour cherry, approximately half of the chosen SNPs were targeted to evenly genetically spanning the *avium* subgenome and half to the *fruticosa* subgenome. Genetic location in the cherry genome of each Stage 2 SNP was estimated by physically anchoring 80 genetically mapped markers (RosCOS, SSR, and CAPS from [6]) to the peach whole genome sequence and then calibrating the genetic location of SNPs between each pair of unambiguously genetically mapped markers according to physical locations. Genetic locations of Stage 2 SNPs in the T×E *Prunus* reference genetic map [3] were also determined using the same approach, anchored by 153 SSR and RFLP markers.

### SNP array evaluation

The RosBREED cherry 6K SNP array v1 was evaluated with panels of sweet cherry (n = 269) and sour cherry (n = 330) germplasm that included a diversity of cultivars, ancestors, founders, and progeny individuals forming a complex pedigree structure linking North American cultivated cherry germplasm for these crops (Tables S3 and S4). The array, employing exclusively Illumina Infinium® II design probes and dual color channel assays (Infinium HD Assay Ultra, Illumina), was used for genotyping following manufacturer recommendations. SNP genotypes were determined using GenomeStudio Genotyping Module Version v2010.3 [19]. All DNA samples were above the GenCall Score threshold of 0.15 and were therefore used in further analyses following the protocols and instructions provided [20]. For sweet cherry, after clustering with the GenomeStudio built-in clustering algorithm, Gentrain2 [21], all SNPs were visually examined for an expected maximum of three clusters (AA, AB, and BB) and then classified as failed, monomorphic, or polymorphic. The AB scores were converted into base pair calls by referencing the Top strand [22]. In contrast to diploid sweet cherry, five genotypes were possible in tetraploid sour cherry for each SNP: AAAA, AAAB, AABB, ABBB, and BBBB. Some sweet cherry accessions (n = 105) were included in the sour cherry GenomeStudio file to help discern the homozygous classes (“AAAA” and “BBBB”) and the balanced heterozygous class (“AABB”). Manual editing was done to check and adjust clusters to the expected genotypic classes following the GenomeStudio polyploid protocol [23]. This recent version of GenomeStudio (Version 2010.3) allows more than three clusters (five in the case of sour cherry) to be manually defined. SNP informativeness in sour cherry was classified using the same criteria as sweet cherry (i.e., failed, monomorphic, or polymorphic) except that a fourth class, termed “unresolved polymorphic”, was used for polymorphic markers that exhibited ambiguous clusters. For each polymorphic SNP (excluding unresolved), MAF among all cultivar and advanced selection panel accessions were determined with GenomeStudio for sweet cherry and manually for sour cherry.

## Results

### SNP detection

A total of 15.7 Gb of sweet cherry and 9 Gb of sour cherry DNA reads were obtained from 308.6 million 80-base reads generated for the 16 sweet cherry and eight sour cherry accessions (Table 1). Excluding ‘Black Republican’ that generated a relatively low number of reads (1.6 million), the total number of sweet cherry reads generated using the Illumina platform averaged 3.1× coverage per accession and ranged from ∼8.8 million reads in ‘Ambrunes’ to 20.8 million reads in ‘Windsor’. The total number of sour cherry reads obtained averaged 1.9× coverage per accession (assuming a genome size of 599 Mb) and ranged from 5.1 million reads in selection 23 23 (13) to 26.1 million reads in selection R1 (1). Approximately 14.1% of the cherry reads were aligned to the Peach v1.0 ‘dhLovell’ whole genome sequence [14]. A total of 1,900,695 SNPs anchored to the peach genome were identified, including polymorphism between peach and cherry, approximately a third of which were within-cherry. Passing the filtering criteria for Stage 1 detection were 1,005,660 SNPs (52.9%), of which 368,948 were within-cherry SNPs – 97,019 within sweet cherry (i.e., polymorphic among sweet cherry detection panel accessions), 320,816 within sour cherry, 63,831 within both crops (included in previous two figures), and 7472 between but not within sweet and sour cherry only (i.e., homozygous in each crop for different alleles) (Figure 1).

### SNP validation

SNP performance via the GoldenGate® assay with a subset of SNPs screened over 160 sweet and sour cherry accessions depended on SNP source, crop screened, and various parameter scores (Table 2). Sweet cherry SNPs screened with sweet cherry accessions performed similarly to sour cherry SNPs screened with sour cherry accessions, with approximately a third of attempted SNPs polymorphic in both cases (Table 2). Evenly spaced and trait locus-targeted SNPs performed similarly for sweet cherry SNPs/accessions. However, for sour cherry the trait locus SNPs were more often polymorphic (50% of SNPs compared to the average of 35%) and failed less often (13% compared to the average of 33%). Accession-specific SNPs performed relatively poorly for both cherry types, as did SNPs in UTRs and intergenic regions. Exonic regions provided the best performing SNPs (42% and 47% polymorphism for sweet and sour cherry, respectively) while intronic SNPs performed only ∼80% as well as exonic SNPs. SNPs with ADT scores of ≥0.90 were the most often polymorphic compared to SNPs with lower ADT scores. Similarly, SNPs with intermediate MAFs (11–40% for sweet cherry and 21–30% for sour cherry) were more often polymorphic than SNPs with extreme MAFs. Sweet cherry SNPs were not as successful on sour cherry accessions (20% polymorphism) while none of the 48 sour cherry SNPs were polymorphic among sweet cherry accessions (Table 2).

**Table 2:** Outcomes of a GoldenGate® validation assay for a set of 144 SNPs screened with 160 cherry accessions to test potential filtering parameters.

		Proportion of SNPs^a^					
		In sweet cherry accessions			In sour cherry accessions		
SNP type	Total^a^	Failed	Monomorphic	Polymorphic	Failed	Monomorphic	Polymorphic
Evenly spaced	80/40	**0.41**/0.28	**0.25**/0.73	**0.34**/0.00	0.64/**0.38**	0.18/**0.30**	0.19/**0.33**
Trait locus	16/8	**0.38**/0.25	**0.31**/0.75	**0.31**/0.00	0.50/**0.13**	0.25/**0.38**	0.25/**0.50**
Accession-specific	22/9	**0.45**/0.22	**0.41**/0.78	**0.14**/0.00	0.50/**0.22**	0.41/**0.67**	0.09/**0.11**
Genic location:							
exonic	38/17	**0.37**/0.29	**0.21**/0.71	**0.42**/0.00	0.47/**0.41**	0.16/**0.12**	0.37/**0.47**
intronic	18/13	**0.33**/0.23	**0.33**/0.77	**0.33**/0.00	0.61/**0.31**	0.22/**0.31**	0.17/**0.38**
UTR	17/9	**0.47**/0.33	**0.29**/0.67	**0.24**/0.00	0.65/**0.22**	0.29/**0.44**	0.06/**0.33**
intergenic	23/9	**0.48**/0.22	**0.26/**0.78	**0.26**/0.00	0.83/**0.33**	0.13/**0.56**	0.04/**0.11**
ADT score:							
<0.8	19/13	**0.47**/0.23	**0.26**/0.77	**0.26**/0.00	0.63/**0.23**	0.21/**0.38**	0.16/**0.38**
0.8–0.9	24/15	**0.42**/0.33	**0.33**/0.67	**0.25**/0.00	0.67/**0.40**	0.25/**0.33**	0.08/**0.27**
≥0.9	53/20	**0.38**/0.25	**0.23**/0.75	**0.40**/0.00	0.58/**0.35**	0.15/**0.25**	0.26/**0.40**
MAF (detection panel)							
1–10%	24/6	**0.42**/0.67	**0.38**/0.33	**0.21**/0.00	0.50/**0.50**	0.33/**0.50**	0.17/**0.00**
11–20%	19/23	**0.32**/0.17	**0.32**/0.83	**0.37**/0.00	0.58/**0.26**	0.21/**0.43**	0.21/**0.30**
21–30%	18/15	**0.39**/0.20	**0.17**/0.80	**0.44**/0.00	0.67/**0.27**	0.06/**0.13**	0.28/**0.60**
31–40%	18/4	**0.33**/0.50	**0.17**/0.50	**0.50**/0.00	0.72/**0.75**	0.06/**0.00**	0.22/**0.25**
41–50%	17/0	**0.59**/na	**0.24**/na	**0.18**/na	0.65/**na**	0.24/**na**	0.12/**na**
Total	96/48	**0.41**/0.27	**0.26**/0.73	**0.33**/0.00	0.61/**0.33**	0.19/**0.31**	0.20/**0.35**

Figures shown in **bold** are for those cases where the crop for which the SNPs were developed matches the crop on which the SNPs were screened.

a Of the 96/48 SNPs examined that targeted sweet cherry/sour cherry.

### SNP final choice

Stage 2 filtering resulted in 31,945 SNPs suitable for the final array. Of these, 28,562 were derived from polymorphism within sweet cherry while the other 3383 were expected to be within sour cherry subgenomes, 1730 indicative of polymorphism within the *avium* subgenome and 1653 within the *fruticosa* subgenome. Partial Stage 2 filtering of the 487 preferentially included SNPs resulted in 413 suitable SNPs.

SNP choices for the 6K array consisted of 4408 sweet cherry SNPs (227 of those being RosCOS SNPs) and 1552 sour cherry SNPs (788 *avium* and 764 *fruticosa*). After a small degree of loss due to technical issues in array manufacturing by Illumina Inc., 5696 SNPs were included on the final array, with 4214 (74%) targeting the sweet cherry genome (221 RosCOS) and 1482 (26%) targeting the sour cherry genome that consisted of 752 for the *avium* subgenome and 730 for *fruticosa* (Table 3). Sweet cherry SNPs targeting each chromosome averaged 527 and ranged from 392 (chromosome 8) to 902 (chromosome 1), which depended on the known genetic length of each chromosome (Table 3). Sour cherry chromosomes of the *avium* subgenome were targeted with an average of 94 SNPs ranging from 66 (chromosome 5) to 164 (chromosome 1) SNPs, while the *fruticosa* chromosomes were targeted with an average of 91 SNPs and ranging from 73 (chromosome 4) to 161 (chromosome 1) SNPs (Table 3). Included RosCOS SNPs were well distributed across the genome, accounting for 3.8 to 6.7% of the sweet cherry SNPs for any given chromosome (Table 3).

**Table 3:** SNP informativeness in sweet cherry for the eight sets of chromosomes based on whether the SNP was derived from polymorphism in sweet cherry or in one of the two sour cherry subgenomes (i.e., *avium* or *fruticosa*).

		Number of SNPs									
Chromosome	SNP source^a^	SNPs chosen^b^	Failed	Monomorphic	Polymorphic	MAF <0.05	MAF 0.05–0.1	MAF 0.1–0.2	MAF 0.2–0.3	MAF 0.3–0.4	MAF 0.4–0.5
1	Sweet	902 (50)	29 (0)	561 (8)	312 (42)	47 (6)	43(3)	72 (4)	62 (9)	33 (7)	55 (13)
	Sour	164/161	6/5	112/155	46/1	10/0	8/1	11/0	8/0	1/0	8/0
2	Sweet	557 (21)	32 (0)	345 (4)	180 (17)	26 (1)	27(0)	30 (6)	53 (4)	20 (0)	23 (6)
	Sour	92/83	9/2	60/80	23/1	5/1	5/0	6/0	2/1	3/0	2/0
3	Sweet	434 (18)	28 (1)	230 (3)	176 (14)	40 (1)	16(4)	45 (2)	39 (6)	18 (0)	18 (1)
	Sour	87/74	5/4	57/68	25/2	7/2	4/0	1/0	5/0	5/0	3/0
4	Sweet	479 (26)	26 (0)	256 (0)	197 (26)	23 (6)	14(0)	37 (2)	42 (6)	44 (7)	37 (5)
	Sour	89/73	8/0	57/72	24/1	4/0	2/0	11/0	4/0	1/0	2/1
5	Sweet	489 (33)	14 (0)	304 (0)	171 (33)	25 (4)	23(5)	23 (4)	28 (3)	42 (7)	30 (10)
	Sour	66/84	6/1	47/83	13/0	4/0	1/0	3/0	1/0	2/0	2/0
6	Sweet	508 (32)	24 (0)	292 (2)	192 (30)	28 (4)	28(1)	34 (5)	33 (8)	38 (8)	31 (4)
	Sour	108/100	1/0	77/94	30/6	10/3	7/2	6/0	0/0	2/1	5/0
7	Sweet	453 (22)	16 (0)	261 (0)	176 (22)	60 (5)	18(1)	37 (2)	23 (5)	22 (6)	16 (3)
	Sour	71/75	2/0	49/72	20/3	7/2	1/0	4/0	4/1	3/0	1/0
8	Sweet	392 (19)	24 (0)	183 (0)	185 (19)	65 (5)	17(1)	23 (0)	25 (2)	25 (5)	30 (6)
	Sour	75/80	6/1	35/72	34/7	7/5	4/1	9/0	6/1	0/0	8/0
Total Sweet		4214 (221)	193 (1)	2432 (17)	1589 (203)	314 (32)	186 (15)	301 (25)	305 (43)	242 (40)	240 (48)
Total Sour		752/730	43/13	494/696	215/21	54/13	32/4	51/0	30/3	17/1	31/1
Grand Total		5696	249	3622	1825	381	222	352	338	260	272

The 269 sweet cherry accessions evaluated are listed in Table S2.

a Numbers of SNPs for the subgenomes of sour cherry are split (/) between *avium* and *fruticosa.*

b Numbers in parentheses are totals for RosCOS SNPs derived from sweet cherry and are included in the first number.

### SNP array evaluation

**Figure 2:**
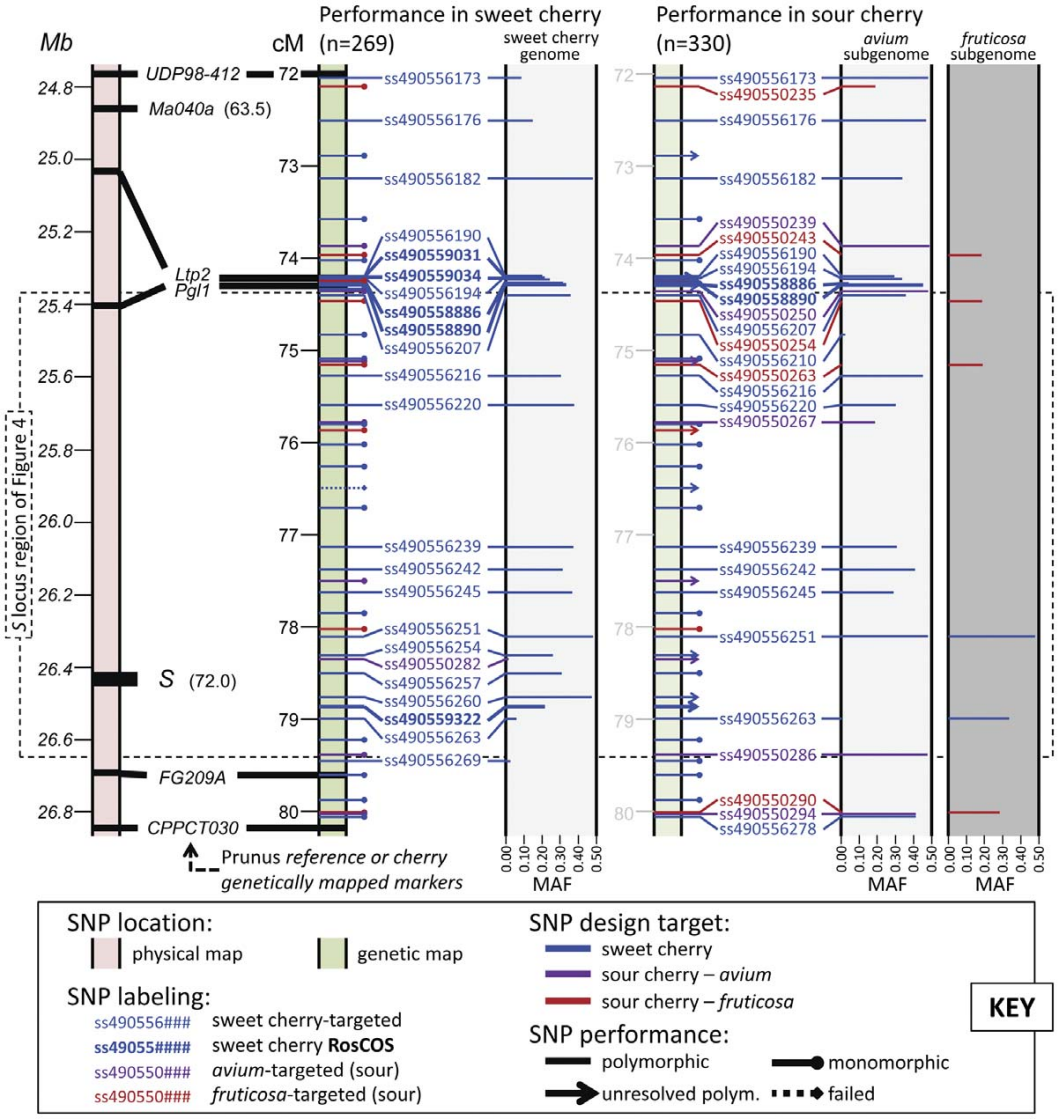
Performance of the RosBREED cherry 6K SNP array v1 for a sample region of the cherry genome. SNPs are shown in a 8.2 cM/2.1 Mb region around the *S* locus at the distal end of *Prunus* LG/chromosome/scaffold 6. Positions of SSR and RFLP markers positioned in the *Prunus* reference map [3] and physical map [14] are indicated. SNP positions are scaled genetically to the 8.2 cM window between reference-mapped markers UDP98-412 and CPPCT030. The *S* locus and Ma040a are also physically positioned, with their genetic locations in the sweet cherry genetic map [4] indicated in parentheses. SNPs designed for sweet cherry that were observed to be polymorphic in sour cherry are assumed here to be polymorphic within the *avium* subgenome of sour cherry except where haplotypes determined for Figure 3 identified polymorphism in both subgenomes (ss490556251) or in *fruticosa* (ss490556263).

Approximately a third of the SNPs on the RosBREED cherry 6K SNP array v1 were observed to be polymorphic in the sweet and sour cherry evaluation panels, and some of the SNPs developed for one crop were successful for the other. Informative SNPs were randomly distributed over the genome such that genetic variation was successfully sampled at medium density in any region of the genome (Figure 2).

A total of 1825 SNPs were polymorphic across the sweet cherry evaluation panel of breeding germplasm, representing 32% of SNPs present on the array (Table 3). Of the remainder, the vast majority (63%) were monomorphic in sweet cherry while 4.7% failed (Table 3). Sweet cherry RosCOS SNPs were highly polymorphic (92% of those tested), dramatically higher than for non-RosCOS sweet cherry SNPs (38%). Of the 4214 SNPs specifically chosen to target the sweet cherry genome, 1589 (38%) were polymorphic for that crop's evaluation panel, while the remaining 236 SNPs polymorphic for sweet cherry were from sour cherry – 221 *avium* (29% of those SNPs) and only 15 *fruticosa* (2.9% of SNPs targeted to that subgenome). A large proportion of sweet cherry SNPs (2432, 58%) were monomorphic in that crop while few failed (193, 5%).

The 1825 SNPs polymorphic in the sweet cherry evaluation panel provided an average physical spacing of approximately 120 kb between SNPs across the sweet cherry genome, considering the peach genome as the proxy for cherry (Table 4). Some gaps were closed by the 236 sour cherry SNPs polymorphic in sweet cherry. Chromosomes 5 and 8 had the smallest average gap length between polymorphic SNPs, each just below 100 kb (Table 4). The largest single gaps between polymorphic SNPs in the sweet cherry genome were on chromosomes 1 and 2 at just under 1.8 Mb each. These largest physical gaps represented estimated genetic gaps of only 0.73 cM (T×E reference map; [3]) or 1.64 cM (sweet cherry RosCOS map; [6]) for chromosome 1 and only 1.28 cM (T×E reference map) or 1.97 cM (cherry RosCOS map) for chromosome 2. The actual largest estimated genetic gap between polymorphic SNPs occurred elsewhere on chromosome 1, at 5.1 cM, the only estimated gap that was >5 cM (Table 4). Average genetic coverage achieved by polymorphic SNPs for the sweet cherry genome was one SNP per 0.29 cM (Table 4).

**Table 4:** Estimated physical and genetic distribution of SNPs of the RosBREED cherry 6K SNP array v1 across sweet cherry chromosomes evaluated for 269 sweet cherry accessions.

Chromosome	Physical			Genetic		
	Average gap (kb)	Largest gap (kb)	Number of gaps >150 kb	Average gap (cM)	Largest gap (cM)	Number of gaps >5 cM
1	38.1^a^ (130.6^b^)	881.3 (1773)	34 (89)	0.09^a^ (0.23^b^)	3.3 (5.1)	0 (1)
2	36.1 (129.3)	705.4 (1740)	28 (55)	0.10 (0.27)	1.1 (2.8)	0 (0)
3	36.7 (107.1)	622.7 (1248)	14 (36)	0.11 (0.25)	1.1 (2.3)	0 (0)
4	46.8 (133.8)	1107.7 (1174)	24 (62)	0.13 (0.27)	4.0 (4.2)	0 (0)
5	28.7 (99.7)	898.0 (1330)	11 (35)	0.11 (0.29)	2.8 (5.0)	0 (0)
6	40.2 (125.4)	396.4 (966.0)	18 (63)	0.17 (0.39)	2.9 (5.0)	0 (0)
7	37.2 (111.8)	387.2 (1065)	19 (46)	0.15 (0.35)	1.1 (3.6)	0 (0)
8	39.7 (96.5)	457.5 (738.0)	20 (49)	0.15 (0.27)	1.6 (1.9)	0 (0)
Genome-wide	37.9 (118.2)	1107.7 (1773)	168 (435)	0.12 (0.29)	4.0 (5.1)	0 (1)

a Based on the 4214 attempted sweet cherry SNPs.

b Based on all 1825 polymorphic SNPs.

Screening of the array with the sour cherry evaluation panel revealed 2058 SNPs (36% of the array) to be polymorphic with their genotypes resolvable (Table 5) – more than for sweet cherry despite only a third as many SNPs being specifically targeted to sour cherry and the accidental use of intergenic SNPs that were expected to be only a quarter as polymorphic as intragenic (Table 2). The 677 polymorphic sour cherry SNPs in this total represented 46% of those targeted to this crop's genome, with slightly more than half being *avium* subgenome SNPs and the rest *fruticosa* (Table 5). Another 1743 SNPs (31%) were polymorphic but could not be resolved into clusters representing the range of expected SNP dosage, while 32% of the array's SNPs were monomorphic for sour cherry and 1.5% failed (Table 5). Of those SNPs targeted to the sour cherry *avium* subgenome, 48% were polymorphic, 41% were unresolved polymorphic, 9% monomorphic, and 1.3% failed for the sour cherry evaluation panel. For *fruticosa* SNPs, these numbers were 43% polymorphic, 39% unresolved polymorphic, 17% monomorphic, and 0.6% failed (Table 5). Sweet cherry RosCOS SNPs were not as successful (57% polymorphism) among sour cherry accessions as they were for sweet cherry accessions but performed better than did other sweet cherry SNPs on sour cherry accessions (33% polymorphism).

**Table 5:** SNP informativeness in sour cherry for the eight sets of chromosomes based on whether the SNP was derived from polymorphism in sweet cherry or in one of the two sour cherry subgenomes (i.e., *avium* or *fruticosa*).

			Number of SNPs									
Chromosome	SNP source^a^	SNPs chosen^b^	Failed	Monomorphic	Unresolved Polymorphic	Polymorphic	MAF <0.05	MAF 0.05–0.1	MAF 0.1–0.2	MAF 0.2–0.3	MAF 0.3–0.4	MAF 0.4–0.5
1	Sweet	902 (50)	8 (0)	364 (11)	211 (8)	319 (31)	14 (2)	7 (3)	43 (5)	28 (6)	88 (6)	139 (9)
	Sour	164/161	1/2	14/18	68/62	81/79	1/0	0/1	3/44	10/13	13/9	54/12
2	Sweet	557 (21)	10 (0)	199 (5)	166 (5)	182 (11)	11 (3)	3 (0)	23 (2)	32 (1)	23 (1)	90 (4)
	Sour	92/83	2/0	12/20	40/26	38/37	3/0	0/0	2/8	2/21	2/3	29/5
3	Sweet	434 (18)	4 (0)	165 (4)	107 (6)	158 (8)	5 (2)	7 (2)	10 (3)	28 (1)	20 (0)	88 (0)
	Sour	87/74	1/2	13/12	33/35	40/25	0/0	0/4	1/7	4/9	4/0	31/5
4	Sweet	479 (26)	9 (0)	176 (0)	141 (9)	153 (17)	7 (4)	5 (1)	11 (3)	38 (5)	20 (2)	72 (2)
	Sour	89/73	1/0	8/15	45/28	35/30	0/0	0/2	2/6	9/17	4/1	20/4
5	Sweet	489 (33)	4 (0)	208 (8)	128 (7)	149 (18)	11 (7)	4 (1)	8 (3)	34 (3)	14 (1)	78 (3)
	Sour	66/84	0/0	4/16	30/36	32/32	0/0	0/1	1/0	1/18	2/3	28/10
6	Sweet	508 (32)	13 (0)	188 (3)	145 (11)	162 (18)	10 (2)	7 (6)	12 (1)	12 (3)	36 (3)	85 (3)
	Sour	108/100	0/0	8/14	43/31	57/55	0/1	0/0	3/25	3/14	3/5	48/10
7	Sweet	453 (22)	6 (0)	184 (5)	113 (3)	150 (14)	12 (5)	2 (0)	10 (2)	21 (2)	20 (3)	85 (2)
	Sour	71/75	2/0	2/16	22/29	45/30	0/0	0/0	3/4	6/18	3/0	33/8
8	Sweet	392 (19)	14 (0)	130 (4)	140 (6)	108 (9)	7 (0)	4 (1)	12 (2)	13 (3)	16 (1)	56 (2)
	Sour	75/80	3/1	9/17	28/36	35/26	3/0	0/1	2/4	2/8	10/4	18/9
Total Sweet		4214 (221)	68 (0)	1614 (40)	1151 (55)	1381 (126)	77 (25)	39 (14)	129 (21)	206 (24)	237 (17)	693 (25)
Total Sour		752/730	10/5	70/128	309/283	363/314	7/1	0/9	17/98	37/118	41/25	261/63
Grand Total		5696	83	1812	1743	2058	85	48	244	361	303	1017

The 330 sour cherry accessions evaluated are listed in Table S3.

a Numbers of SNPs for the subgenomes of sour cherry are split (/) between *avium* and *fruticosa.*

b Numbers in parentheses are totals for RosCOS SNPs derived from sweet cherry and are included in the first number.

The 2058 SNPs polymorphic in the sour cherry evaluation panel provided an average physical spacing of 124 kb between SNPs across the sour cherry *avium* subgenome and 666 kb across the sour cherry *fruticosa* subgenome (Table 6). As sweet cherry SNPs polymorphic in sour cherry were assumed in these calculations to be within the *avium* subgenome, this subgenome therefore had almost five times greater density of polymorphic SNPs than the *fruticosa* subgenome. Sour cherry's *avium* chromosome 7 had the largest single gap between polymorphic SNPs of 1.9 Mb (Table 6), corresponding to an estimated 1.50 cM (T×E reference map) or 5.92 cM (cherry RosCOS map) in this region. For the *fruticosa* subgenome, the largest physical gap, of 8.3 Mb, was on chromosome 2 (Table 6). Such a gap in this region corresponded to an estimated 9.55 cM (T×E reference map) or 6.62 cM (sweet cherry RosCOS map). However, the largest estimated genetic gaps for each subgenome were elsewhere in the genome, on chromosome 6 for *avium* (7.3 cM) and chromosome 7 for *fruticosa* (11.2 cM) (Table 7). Although the SNPs specifically targeting the sour cherry *avium* and *fruticosa* subgenomes resulted in average densities of one polymorphic SNP per 1.3 and 1.5 cM (T×E reference map), respectively, sweet cherry SNPs polymorphic in this other cherry crop improved genetic resolution for *avium* to one SNP per 0.29 cM, assuming all such SNPs were indeed within this subgenome (Table 7). Only two gaps between polymorphic SNP markers for the *avium* subgenome and 13 gaps for *fruticosa* were >5 cM (Table 7).

**Table 6:** Estimated physical distribution of SNPs of the RosBREED cherry 6K SNP array v1 across sour cherry chromosomes evaluated for 330 sour cherry accessions.

Chromosome	*avium* subgenome			*fruticosa* subgenome		
	Average gap (kb)	Largest gap (kb)	Number of gaps >150 kb	Average gap (kb)	Largest gap (kb)	Number of gaps >150 kb
1	282.0^a^ (116.8)^b^	2259 (1384)	111 (92)	288.6 (588.2)	2812 (4166)	107 (66)
2	286.3 (120.1)	1472 (1548)	53 (48)	310.7 (696.9)	2442 (8302)	49 (28)
3	249.2 (109.8)	1073 (1207)	54 (37)	291.5 (862.9)	1545 (3428)	48 (21)
4	333.6 (158.5)	1649 (1281)	73 (64)	401.2 (884.4)	3077 (8057)	56 (27)
5	276.1 (101.3)	2438 (973.5)	26 (30)	215.9 (565.3)	1755 (2701)	43 (22)
6	262.4 (131.3)	1611 (1267)	72 (61)	282.0 (509.4)	2215 (2990)	67 (46)
7	308.9 (114.1)	1106 (1900)	51 (43)	290.3 (714.5)	2113 (4204)	47 (26)
8	289.3 (151.9)	1642 (925.6)	47 (43)	266.8 (810.2)	2024 (4350)	51 (25)
Genome-wide	284.8 (123.7)	2438 (1900)	487 (418)	291.2 (665.5)	3077 (8302)	468 (261)

a Based on the 1482 attempted sour cherry SNPs (752 for the *avium* subgenome and 730 for *fruticosa*).

b Based on all 2058 polymorphic SNPs; for the *avium* subgenome, this calculation included all sweet cherry SNPs polymorphic for sour cherry.

**Table 7:** Estimated genetic distribution of SNPs of the RosBREED cherry 6K SNP array v1 across sour cherry chromosomes evaluated for 330 sour cherry accessions.

Chromosome	*avium* subgenome			*fruticosa* subgenome		
	Average gap (cM)	Largest gap (cM)	Number of gaps >5 cM	Average gap (cM)	Largest gap (cM)	Number of gaps >5 cM
1	0.48^a^ (0.20)^b^	6.1 (5.8)	1 (1)	0.49 (0.99)	4.7 (5.6)	0 (2)
2	0.52 (0.24)	2.7 (1.5)	0 (0)	0.56 (1.26)	2.8 (9.6)	0 (1)
3	0.54 (0.25)	3.4 (2.0)	0 (0)	0.62 (1.84)	3.3 (7.4)	0 (2)
4	0.60 (0.30)	4.5 (4.0)	0 (0)	0.73 (1.74)	4.2 (4.2)	0 (0)
5	0.74 (0.28)	5.8 (5.0)	1 (1)	0.59 (1.52)	5.8 (6.6)	1 (2)
6	0.76 (0.40)	3.6 (7.3)	0 (0)	0.81 (1.48)	5.2 (8.3)	1 (3)
7	0.92 (0.35)	3.2 (4.1)	0 (0)	0.87 (2.13)	4.8 (11.2)	0 (2)
8	0.82 (0.43)	4.3 (2.7)	0 (0)	0.72 (2.10)	2.5 (5.8)	0 (1)
Genome-wide	0.64 (0.29)	6.1 (7.3)	2 (2)	0.65 (1.50)	5.8 (11.2)	2 (13)

a Based on the 1482 attempted sour cherry SNPs (752 for the *avium* subgenome and 730 for *fruticosa*).

b Based on all 2058 polymorphic SNPs; for the *avium* subgenome, this calculation included all sweet cherry SNPs polymorphic for sour cherry.

**Figure 3:**
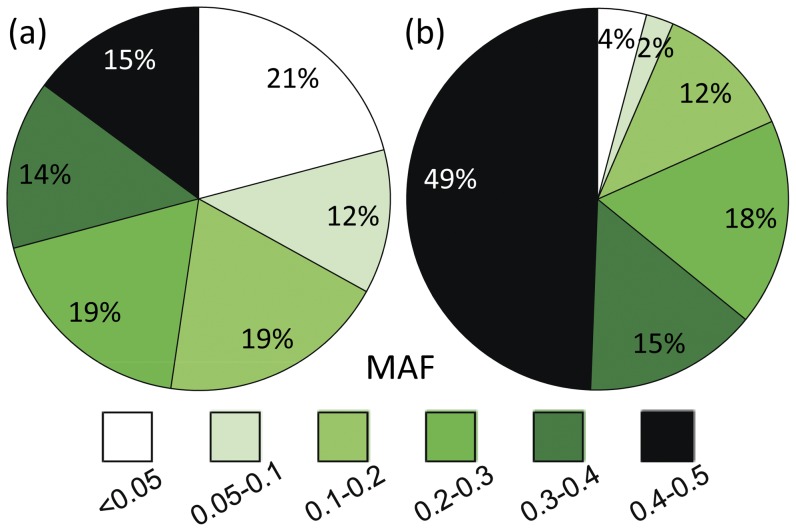
Minor allele frequency (MAF) of polymorphic cherry SNPs. MAF was determined in sweet cherry (n = 50) and sour cherry (n = 37) evaluation panels for 1825 and 2058 polymorphic SNPs, respectively.

Half of the polymorphic SNPs in sweet cherry had a MAF >0.2, while a third were <0.10; in contrast, half of the polymorphic SNPs in sour cherry were >0.4 MAF and only 6% were <0.10 (Figure 3). Of the 1036 SNPs polymorphic in both crops, there was little correlation between MAFs (r = −0.13). This observation held considering just the sweet cherry SNPs polymorphic in both crops (r = −0.11, n = 919), the sour cherry *avium* SNPs (r = −0.22, n = 110), or the sour cherry *fruticosa* SNPs (r = not applicable, n = 7). Average heterozygosity in the 16 sweet cherry accessions of the detection panel for sweet cherry-targeted SNPs was 0.118 (s.d. 0.013) and was similar for all other non-seedling accessions of the evaluation panel (average 0.110, s.d. 0.012). For sweet cherry accessions such as the MIM series and ‘Walpurgis’ with no known pedigree connections to detection panel accessions, heterozygosity was similar (results not shown). For all SNPs on the array, heterozygosity of sweet cherry accessions averaged 0.103 and ranged from 0.075 to 0.124.

**Figure 4:**
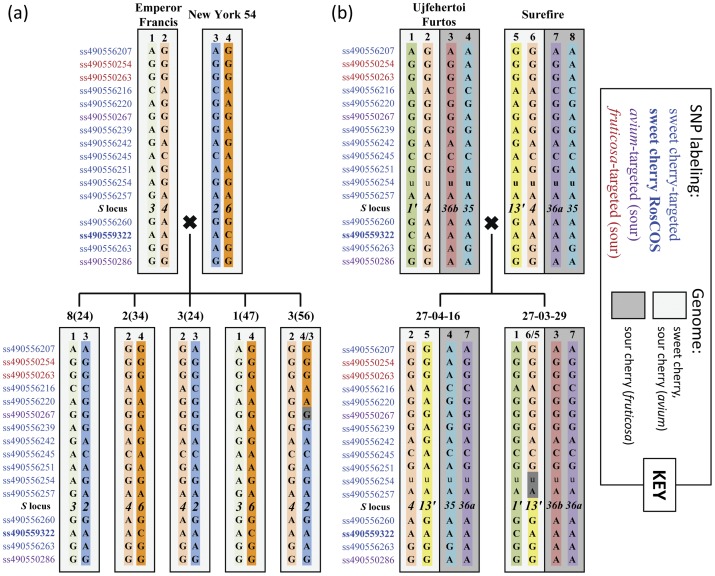
Reconstruction of a ∼1.2 Mb region spanning the self-incompatibility *S* locus and its inheritance in cherry. (a) Sweet cherry, with four parental haplotypes (1–4). (b) Sour cherry, with eight parental haplotypes (1–8). Identical haplotypes have the same background colors. Haplotypes are shown for five sweet cherry and two sour cherry seedlings. Monomorphic SNPs within cross-over regions are highlighted in grey. Genotypes indicated as “u” are for an unresolved polymorphic SNP in sour cherry.

In a ∼1.2 Mb region spanning the self-incompatibility *S* locus on *Prunus* LG6 for the sweet cherry cross ‘New York 54’ × ‘Emperor Francis’, offspring representing the four possible non-recombinant haplotypes were identified (Figure 4A). One individual, 3 (56), was identified to have resulted from a recombination of ‘New York 54’ haplotypes, with the recombination site localized to a ∼400 kb, ∼1.5 cM interval (T×E reference map). For this sweet cherry cross, all sour cherry-derived SNPs were homozygous in this region. SNP haplotypes constructed for the same *S* locus region could also be followed from parents to offspring of the sour cherry cross ‘Ujfehertoi Furtos’ × ‘Surefire’ (Figure 4B). The two sour cherry cultivars shared two of their four *S*-alleles (*S_4_* and *S_35_*) for which their SNP haplotypes were identical across the examined region; the *S_4_* haplotype was also shared with the sweet cherry cultivar Emperor Francis (Figure 4A and 4B). A recombination between the *S_4_* and *S_13′_* haplotypes of ‘Surefire’ was identified in progeny individual 27-03-29, localized to a ∼100 kb, ∼0.5 cM interval. Nine of the 11 sweet cherry-derived SNPs were informative in this cross as the SNPs were heterozygous within at least one of the subgenomes. In this sour cherry cross, three of four sour cherry-targeted SNPs were heterozygous only in the *fruticosa* subgenome while polymorphism observed for the fourth, ss490550286, was between but not within the *avium* and *fruticosa* subgenomes (Figure 4B). One sweet cherry-targeted SNP, ss490556254, was polymorphic in the sour cherry cross; however, dosage could not be resolved. Another sweet cherry-targeted SNP, ss490556251, was polymorphic in both the *avium* and *fruticosa* subgenomes (Figure 4B, also indicated in Figure 2).

### Public availability of SNP information

All 5696 SNPs on the RosBREED cherry 6K SNP array v1 (4214 sweet cherry and 1482 sour cherry; Tables S5, S6, S7) were deposited in NCBI's dbSNP repository [24] available at www.ncbi.nlm.nih.gov/projects/SNP and also at the Genome Database for Rosaceae (GDR; [25]) at www.rosaceae.org. GDR provides a downloadable Excel file on the peach genome project page containing the genomic locations, flanking sequence, and web links to a GBrowse viewer for these cherry SNPs on the peach genome.

## Discussion

The RosBREED cherry 6K SNP array v1was determined to be useful for various genetics studies of cultivated cherry, despite the use of a relatively low number of detection panel accessions each sequenced at low depth. The number of SNP detection panel accessions, 16 for sweet cherry and eight for sour cherry, achieved a total depth of genome coverage (46.3× for sweet cherry and 15× for sour cherry) that was less than that of recent SNP detection and array development for peach and apple, two of cherry's rosaceous relatives. The recently developed International Peach SNP Consortium peach 9K SNP array v1 used 56 accessions that achieved 118× total genome coverage and for which 84.3% of included SNPs were informative when evaluated on diverse breeding germplasm [13]. The 27 accessions used for the International RosBREED SNP Consortium apple 8K SNP array v1 achieved 89× total genome coverage and 70.6% of SNPs were informative [7]. However, sequencing depth per accession was similar among all these efforts, averaging 3.1× genome coverage per accession for sweet cherry and 2.0× for sour cherry (Table 1), 2.2× for peach [13], and 3.3× for apple [7]. Sour cherry coverage was comparable because the sequencing was performed at twice the depth of sweet cherry. As the proportion of informative cherry SNPs was only half that of peach and apple (only 38% and 46% of those targeted to sweet cherry and sour cherry breeding germplasm, respectively), the relative limitation of the cherry SNP detection panel was either the number or relevance of detection panel accessions. The number of accessions was a compromise based on the available budget and intended allocation of array attention of 75% to sweet cherry and 25% to sour cherry, utilizing state-of-the-art NGS technology (Illumina GA II). Because of the intended use of the array in characterizing genetic variation in cultivated cherry, the few accessions of the detection panel were carefully chosen to efficiently represent cherry breeding germplasm. For both sweet and sour cherry, choices were based on prior knowledge of geographic origin, pedigree, SSR and RosCOS SNP diversity (sweet cherry, [6]) or isozyme diversity (sour cherry, [26]), and the potential to confer disease resistance [27], [28]. The detection panel choice appears to have been suitable because the array performed as well on the original detection panel accessions as on other breeding germplasm, including on material unconnected by pedigree. With the large number of SNPs on the array, the observed levels of polymorphism and heterozygosity translate to an unprecedented resolution for assaying genetic variation in cherry. In sweet cherry, for example, the array is expected to reveal 1500–2000 polymorphic genome-wide SNPs for any given set of cultivars, and 400–700 SNPs heterozygous within any given cultivar, from our observations of polymorphism and heterozygosity in the sweet cherry evaluation panel. This estimate is supported by [29] who reported 515 to 634 SNPs heterozygous for each of four cultivars when this 6K array was used as the basis for the two highest density genetic maps to date of a *Prunus* species. Therefore, the set of SNPs on the array developed from a small but diverse set of breeding-relevant accessions is expected to be generally informative for cherry germplasm in cultivation and in breeding programs.

The proportion of polymorphic markers verified in the sour cherry germplasm was nearly double that found in the sweet cherry set. However, dosage could be resolved for only approximately half these SNP markers. Therefore, despite having twice the number of chromosomes, and using intergenic rather than intragenic SNPs, the final number of resolvable polymorphic sour cherry SNPs was just slightly more than that for sweet cherry, 2058 versus 1825. Given that only 314 of these markers were chosen to putatively target the *fruticosa* subgenome, it is suspected that the sour cherry *avium* subgenome will have significantly denser marker coverage than the *fruticosa* subgenome. However, it is probable that some of the sweet cherry SNPs that were polymorphic in sour cherry were within the *fruticosa* subgenome, given that 215 of the 236 sour cherry SNPs polymorphic in sweet cherry were *avium*-targeted.

Here, a comparative genomics approach relied heavily on the peach reference genome sequence for SNP detection (aligning sequence reads) and for final choice of SNPs for the array (evenly spanning the “cherry” genome rather than random positions). Cherry and peach belong to the same genus, with the same chromosome number and apparent genetic co-linearity [3], [4], [6]. However, the two crops are in divergent subgenera [30] and not cross-compatible; therefore, there may be significant genomic differences at the micro-syntenic and DNA sequence levels. The latter may account for the low proportion (14.1%) of raw cherry reads that could be aligned to the peach genome given the strict alignment criteria used here, such that only sequences from the most conserved regions between cherry and peach genomes were interrogated for SNPs. If the 86% of unaligned cherry reads were due to substantial sequence-level divergence in localized regions (spanning a few cM or more), the array will be non-informative for such regions of the cherry genome. Similarly, large-scale deletions in the peach genome compared to cherry would result in non-sampled regions. If such deletions occurred at chromosome ends, even the successful genetic mapping of these cherry SNPs would not detect the missing regions. Translocations between the two subgenera occurring between previously genetically mapped markers and on the scale of up to millions of base pairs or several cM are not expected to affect the array's genome coverage. As genetic locations used to evenly space SNPs across the “cherry” genome were based on RosCOS SNPs previously genetically mapped in the sweet cherry genome, SNP spacing should not be affected by any differences in recombination rates between peach and cherry.

The high degree of observed monomorphism appears to be due to total sequencing depth of the SNP detection panel. Of the sweet cherry SNPs that did align to the peach genome and passed the various filters to be included on the array, the large proportion (58%) of monomorphism and low failure rate (5%) in sweet cherry accessions indicates that sequences were accurately aligned to unique locations in the peach genome but that detected SNPs were false positives. While a low failure rate (1%) for sour cherry SNPs in sour cherry accessions also indicates successful sequence alignment to the peach genome, the relatively low rate of monomorphism (13%) suggests that most of the originally detected sour cherry SNPs were true SNPs for that crop. However, if unresolved polymorphic sour cherry SNPs were actually monomorphic because their variation in genotype clustering (apparent polymorphism) was due to sequence variation in flanking sequences, then such an adjusted monomorphism rate for sour cherry (53%) would be similar to that of sweet cherry. For peach and apple, monomorphism and failure rates were lower, totaling 16% for peach [13] and 28% for apple [7], probably due the greater total depth of detection panel sequencing for those crops as discussed above. In fact, there is a strong linear relationship between polymorphism rate (P) and total sequencing depth (D) among the three diploid crops of apple, peach, and sweet cherry (P = 0.75D, R^2^ = 0.96), suggesting that, using similar filtering parameters among these crops, false positives due to sequencing errors and failure due to undetected polymorphism in SNP-flanking sequences could be effectively avoided with a total sequencing depth of at least 133×. This prediction is an expected consequence of the pseudo-random genomic sampling that underlies shotgun sequencing approaches; detection of individual SNPs requires deep sequencing at individual bases and detection of SNPs genome-wide requires broad sequencing over the genome space. Therefore sufficiently deep sequencing is required to identify not only individual SNPs that are potentially of value for downstream assays but to also discover nearby SNP-flanking polymorphisms that can cause SNP assay failures.

The above considerations suggest some alternative array development strategies. The number of polymorphic SNPs in the cherry array could have been doubled by focusing available resources for SNP detection on one crop or the other (sweet or sour cherry) to double the sequencing depth for that crop. Due to a shared genomic background, such a single cherry crop array would have been useful for the other crop to a certain extent. Following simple extrapolations from our observed cross-species results (Tables 3 and 5), a 6K sweet cherry array based on a detection panel of only sweet cherry accessions (achieving an estimated 100× total genome coverage and 75% polymorphism rate) would be expected to provide polymorphic SNPs of ∼4300 for sweet cherry and ∼1900 for sour cherry highly skewed to the *avium* subgenome. Focusing on sour cherry SNP detection instead, a 6K sour cherry array would be expected to provide polymorphic SNPs of ∼4300 for sour cherry (equally distributed over both subgenomes) but only ∼900 for sweet cherry. Considering that SNP polymorphism was desired for both crops, both hypothetical scenarios are inferior to the actual dual-crop SNP detection strategy employed that achieved polymorphism of 1825 SNPs in sweet cherry and 2058 SNPs in sour cherry. However, the 2058 SNPs polymorphic in sour cherry are likely to be skewed somewhat toward the *avium* subgenome because most were developed for sweet cherry (*P. avium*). A better strategy for the dual-crop array would have been to strongly bias the choice of sour cherry SNPs to the *fruticosa* subgenome; in hindsight, targeting all sour cherry SNPs to the *fruticosa* subgenome would have better balanced subgenome coverage.

The RosBREED cherry 6K SNP array v1 appears to be informative beyond just cherry breeding and cultivated germplasm. The *avium* subgenome of sour cherry is believed to represent a subset of *P. avium* species diversity that pre-dates modern cultivated sweet cherry [2]. Revealingly, 29% of *avium*-subgenome sour cherry SNPs (215) were polymorphic for sweet cherry and 33% of sweet cherry SNPs (1380) were polymorphic for sour cherry. Therefore, ∼30% of SNPs on the array are expected to be polymorphic for any diverse set of sweet cherry germplasm, including wild populations. A similar transferability rate may extend to wild *P. cerasus* populations for the 1482 sour cherry SNPs. Transferability of SNP polymorphism between the distinct *P. avium* and *P. fruticosa* species (effectively targeted with 4966 and 730 SNPs on the array, respectively) is estimated to be only a tenth of the transferability within *P. avium* given that 10% of the sour cherry SNPs polymorphic for sweet cherry accessions were originally developed for *fruticosa*. The ∼200 RosCOS SNPs performed exceptionally well because of their previous validation in cherry germplasm [6]. These RosCOS markers provide a valuable tool for comparative genomics in Rosaceae such as comparing functional genetic variation across genera. More than 100 RosCOS SNPs are expected to be polymorphic in apple from the 128 such markers included on the apple 8K array [7]. While only 14 RosCOS SNPs were polymorphic for peach [13], hundreds of other markers polymorphic in both peach and cherry provide sufficient anchors for comparative genomics within the *Prunus* genus [3], [4].

Close examination of haplotype segregation for a 16-SNP region at the *S* locus (Figure 4) indicated that use of the array should enable monitoring of all recombination events in cherry germplasm for any two subsequent generations examined at a time. Unique haplotypes and recombinations between them were successfully detected and localized for both sweet and sour cherry around the *S* locus. The unprecedented resolution of genetic variation in cherry germplasm revealed by genome-scanning with the array opens the door to fine-scale QTL dissection and other linkage-based analyses, identification of incorrect pedigree records, and deduction of identity by descent for chromosomal segments across the genome. In the region examined, common haplotypes, indicating strong evidence for common ancestry, were identified both within a crop (*S_4_* and *S_35_* in sour cherry) and between the two crops (*S_4_*). The *S_1′_*, *S_4_*, and *S_13′_* alleles of sour cherry are known to be *avium*-derived as these *S* alleles are present in sweet cherry germplasm [31], [32], [33]. For example, the *S_4_* allele of the sweet cherry parent ‘Emperor Francis’ was observed to be identical to the *S_4_* haplotype present in both sour cherry parents. In contrast, the *S_35_*, *S_36a_*, and *S_36b_* alleles are considered to be *fruticosa-*derived as they have not been observed in sweet cherry germplasm [34]. Reconstruction of the two sour cherry subgenomes that is ongoing using the SNP data and a linkage mapping approach is predicted to be complicated by the lack of balanced subgenomes within sour cherry germplasm as sour cherry is known to be a segmental allotetraploid [34], [35]. Initial insight into reconstruction of sour cherry subgenomes was illustrated with the identification of seven sour cherry *S*-allele haplotypes. However, as the *S* locus region is known to be subject to recombinational suppression and as a result, a site for the accumulation of polymorphism, this level of subgenome-balanced polymorphism should not be extrapolated genome-wide.

## Conclusion

The cherry SNP array described here will foster genetics studies in the Rosaceae and help bridge the gap between genomics and breeding in cherry because breeding germplasm was the basis of detected SNPs and SNP choices of the final array. The RosBREED cherry 6K SNP array v1 is commercially available from Illumina and we expect that it will be used worldwide for genetic studies in cherry and related species. The SNP markers included in the cherry 6K Illumina arrays are available for download in Excel format and viewable in GBrowse at the Genome Database for Rosaceae (GDR; http://www.rosaceae.org).

## Supporting Information

Table S1 Sweet cherry validation panel of 79 sweet cherry accessions, used in the GoldenGate® assay for validation of detected SNPs.
(DOCX)

Table S2 Sour cherry validation panel of 81 sour cherry accessions, used for validation of detected SNPs.
(DOCX)

Table S3 Sweet cherry evaluation panel of 269 sweet cherry accessions, used in the Infinium® II assay for SNP array evaluation.
(DOCX)

Table S4 Sour cherry evaluation panel of 330 sour cherry accessions, used for SNP array evaluation.
(DOCX)

Table S5 All SNPs of the RosBREED cherry 6K SNP array v1. Information includes physical and genetic positions, targeted genome, and performance outcomes for sweet cherry (n = 269) and sour cherry (n = 330) evaluation panels.
(XLSX)

Table S6 Markers of the *Prunus* reference T×E genetic map [3] used for estimating genetic positions of cherry 6K array SNPs.
(XLSX)

Table S7 Markers of the sweet cherry genetic map [6] used for estimating genetic positions of cherry 6K array SNPs.
(XLSX)
