# Supplementary material for: Development and Evaluation of a Genome-Wide 6K SNP Array for Diploid Sweet Cherry and Tetraploid Sour Cherry
Source: PLoS One. 2012 Dec 20;7(12):e48305. doi: 10.1371/journal.pone.0048305 (PMC3527432; doi:10.1371/journal.pone.0048305)
Supplement: Table S1 — Sweet cherry validation panel of 79 sweet cherry accessions, used in the GoldenGate® assay for validation of detected SNPs. (DOCX) [file pone.0048305.s001.docx]

Table S1: Sweet cherry validation panel of 79 sweet cherry accessions, used in the GoldenGate® assay for validation of detected SNPs.

| **Accession** | **Mother** | **Father** |
| --- | --- | --- |
| 4.10.15-001 | Lapins | Regina |
| 4.14.17-001 | Rainier | Sunburst |
| 8011-3 | -^1^ | - |
| 9819-005 | PMR-1 | Van |
| 9819-031 | PMR-1 | Van |
| AA | PMR-1 | Rainier |
| Ambrunes | - | - |
| BB | PMR-1 | Rainier |
| Black Republican | - | - |
| Brooks | Rainier | Early Burlat |
| Cashmere | Stella | Early Burlat |
| CC | PMR-1 | Rainier |
| Chelan | Stella | Beaulieu |
| Chinook | Bing | Gil-Peck |
| Cowiche | PC7147-4 | PC7146-11 |
| Early Burlat | - | - |
| EE | PMR-1 | Rainier |
| Emperor Francis | - | - |
| GG | Rainier | PMR-1 |
| Gil-Peck | Napoleon | Giant |
| Glacier | Stella | Early Burlat |
| Index | Stella | - |
| JJ | PMR-1 | Rainier |
| Kiona | Glacier | Cashmere |
| Kordia | - | - |
| Krupnoplodnaya | Drogana Zholtaya | Valeriy Chkalov |
| Lambert | Napoleon | Black Heart |
| Lapins | Van | Stella |
| MIM 3 | - | - |
| MIM 13 | - | - |
| MIM 17 | - | - |
| MIM 20 | - | - |
| MIM 23 | - | - |
| Moreau | - | - |
| Napoleon | - | - |
| PMR-1 | - | - |
| Rainier | Bing | Van |
| Regina | Schneiders | Rube |
| Sam | Windsor | - |
| Sato Nishiki | Governor Wood | Napoleon |
| Schmidt | - | - |
| Schneiders | - | - |
| Selah | P8-9 | Stella |
| Stella | Lambert | JI 2420 |
| Summit | Van | Sam |
| Sunburst | Van | Stella |
| Sweetheart | Van | Newstar |
| Tieton | Stella | Early Burlat |
| Ulster | Schmidt | Lambert |
| Van | Empress Eugenie | - |
| Venus | Hedelfingen | Windsor |
| Vic | Bing | Schmidt |
| Walpurgus | - | - |
| Windsor | - | - |
| Family A (n=6) | Lapins | Chelan |
| Family B (n=8) | New York 54 | Emperor Francis |
| Family C (n=2) | Sweetheart | Ambrunes |
| Family D (n=6) | Sweetheart | Regina |
| Family E (n=2) | Sweetheart | Tieton |

^1^Designates unknown parent
