# Supplementary material for: Development and Evaluation of a Genome-Wide 6K SNP Array for Diploid Sweet Cherry and Tetraploid Sour Cherry
Source: PLoS One. 2012 Dec 20;7(12):e48305. doi: 10.1371/journal.pone.0048305 (PMC3527432; doi:10.1371/journal.pone.0048305)
Supplement: Table S2 — Sour cherry validation panel of 81 sour cherry accessions, used for validation of detected SNPs. (DOCX) [file pone.0048305.s002.docx]

Table S2: Sour cherry validation panel of 81 sour cherry accessions, used for validation of detected SNPs.

| **Accession** | **Mother** | **Father** |
| --- | --- | --- |
| 25 02 (29) | Rheinische Schattenmorelle | Erdi Botermo |
| 25 14 (20) | English Morello | - |
| Almaz R1(1) | Almaz | - |
| Englaise Timpurii | - | - |
| M172 | Pandy 38 | Eugenia |
| Montmorency | - | - |
| Nana | Crisana | - |
| New York 54 | - | - |
| Pitic de Iasi | - | - |
| Rheinische Schattenmorelle | Landrace A | - |
| Surefire | Borchert Black Sour | New York 6935 |
| Újfehértói Fürtös^2^ | - | - |
| 23 23 (13) | 148-1 | - |
| Family A (n=15) | 25 14 (20) | 25 02 (29) |
| Family B (n=15) | M172 | 25 02 (29) |
| Family C (n=2) | Montmorency | 23 23 (13) |
| Family D (n=15) | Rheinische Schattenmorelle | Englaise Timpurii |
| Family E (n=3) | Újfehértói Fürtös | 23 23 (13) |
| Family F (n=18) | Újfehértói Fürtös | Surefire |

^1^Designates unknown parent

^2^Mutant of Pandy
