# Supplementary material for: Development and Evaluation of a Genome-Wide 6K SNP Array for Diploid Sweet Cherry and Tetraploid Sour Cherry
Source: PLoS One. 2012 Dec 20;7(12):e48305. doi: 10.1371/journal.pone.0048305 (PMC3527432; doi:10.1371/journal.pone.0048305)
Supplement: Table S3 — Sweet cherry evaluation panel of 269 sweet cherry accessions, used in the Infinium® II assay for SNP array evaluation. (DOCX) [file pone.0048305.s003.docx]

Table S3: Sweet cherry evaluation panel of 269 sweet cherry accessions, used in the Infinium® II assay for SNP array evaluation.

| **Accession** | **Mother** | **Father** |
| --- | --- | --- |
| AA | PMR-1 | Rainier |
| Ambrunes | -^1^ | - |
| BB | PMR-1 | Rainier |
| Benton | Stella | Beaulieu |
| Black Republican | - | - |
| Brooks | Rainier | Early Burlat |
| Cashmere | Stella | Early Burlat |
| CC | PMR-1 | Rainier |
| Chelan | Stella | Beaulieu |
| Chinook | Bing | Gil-Peck |
| Cowiche | PC7147-4 | PC7146-11 |
| DD | PMR-1 | Rainier |
| Early Burlat | - | - |
| EE | PMR-1 | Rainier |
| Emperor Francis | - | - |
| GG | Rainier | PMR-1 |
| Gil-Peck | Napoleon | Giant |
| Glacier | Stella | Early Burlat |
| JJ | PMR-1 | Rainier |
| Kiona | Glacier | Cashmere |
| Lambert | Napoleon | Black Heart |
| Lapins | Stella | Van |
| MIM 3 | - | - |
| MIM 13 | - | - |
| MIM 17 | - | - |
| MIM 20 | - | - |
| MIM 23 | - | - |
| Napoleon | - | - |
| New York 54 | - | - |
| PMR-1 | - | - |
| Rainier | Bing | Van |
| Regina | Schneiders | Rube |
| Sam | Windsor | - |
| Sato Nashiki | Governor Wood | Napoleon |
| Schmidt | - | - |
| Schneiders | - | - |
| Selah | P8-79 | Stella |
| Stella | Lambert | JI 2420 |
| Summit | Van | Sam |
| Sweetheart | Van | Newstar |
| Tieton | Stella | Early Burlat |
| Ulster | Schmidt | Lambert |
| Van | Empress Eugenie | - |
| Vic | Bing | Schmidt |
| Walpurgis | - | - |
| Windsor | - | - |
| FR2T030 | Rainier | Sunburst |
| FR2T063 | Lapins | Regina |
| FR3T005 | - | - |
| FR3T043 | - | - |
| FR3T070 | Selah | Ambrunes |
| FR17T059 | - | - |
| Family A (n=6) | BB | MIM 17 |
| Family B (n=3) | Benton | Ambrunes |
| Family C (n=2) | Benton | Regina |
| Family D (n=2) | Bing | - |
| Family E (n=12) | Bing | PMR-1 |
| Family F (n=7) | CC | MIM 17 |
| Family G (n=12) | Cowiche | Regina |
| Family H (n=7) | Cowiche | Summit |
| Family I (n=13) | DD | MIM 17 |
| Family J (n=10) | EE | MIM 17 |
| Family K (n=4) | GG | MIM 17 |
| Family L (n=10) | Kiona | Chelan |
| Family M (n=2) | Kordia | - |
| Family N (n=19) | Lapins | Ambrunes |
| Family O (n=14) | Lapins | Chelan |
| Family P (n=2) | Lapins | - |
| Family Q (n=6) | Lapins | Tieton |
| Family R (n=18) | New York 54 | Emperor Francis |
| Family S (n=4) | PMR-1 | Van |
| Family T (n=9) | Rainier | Ambrunes |
| Family U (n=4) | Rainier | Benton |
| Family V (n=11) | Rainier | PMR-1 |
| Family W (n=13) | Rainier | Regina |
| Family X (n=3) | Sweetheart | - |
| Family Y (n=22) | Sweetheart | Regina |

^1^Designates unknown parent
