# Supplementary material for: Development and Evaluation of a Genome-Wide 6K SNP Array for Diploid Sweet Cherry and Tetraploid Sour Cherry
Source: PLoS One. 2012 Dec 20;7(12):e48305. doi: 10.1371/journal.pone.0048305 (PMC3527432; doi:10.1371/journal.pone.0048305)
Supplement: Table S4 — Sour cherry evaluation panel of 330 sour cherry accessions, used for SNP array evaluation. (DOCX) [file pone.0048305.s004.docx]

Table S4: Sour cherry evaluation panel of 330 sour cherry accessions, used for SNP array evaluation.

| **Accession** | **Mother** | **Father** |
| --- | --- | --- |
| 25 02 (29) | Rheinische Schattenmorelle | Erdi Botermo |
| 25 08 (46) | Meteor | I 21 (17) |
| 25 14 (20) | English Morello | -^1^ |
| 26e 11 (27) | II 03 (11) | Csengodi |
| 26e 17 (29) | II 08 (16) | I 13 (61) |
| 27 08 (30) | Meteor | Erdi Jubileum |
| 27 10 (51) | II 07 (14) | Erdi Jubileum |
| 27 13 (65) | Újfehértói Fürtös | Rheinische Schattenmorelle |
| 27 27 (44) | 25 14 (42) | I 13 (61) |
| 27e 15 (10) | M172 | - |
| 27e 15 (42) | M172 | - |
| Crisana | Landrace B | - |
| Csengodi | - | - |
| Englaise Timpurii | - | - |
| English Morello | - | - |
| Erdi Botermo | Pandy 38 | Nagy Angol |
| Erdi Jubileum | Pandy 38 | Eugenia |
| Favorit | Pandy 38 | Montreulli |
| I 13 (61) | Northstar | Kansas Sweet |
| I 63 (05) | Nefris | Meteor |
| II 07 (14) | English Morello | Meteor Korai |
| II 06 (27) | English Morello | Meteor |
| III 18 (12) | HY38-13 | - |
| Korai Pipacs Meggy | Pandy 38 | Csaszar |
| M172 | Pandy 38 | Eugenia |
| Meteor | Montmorency | - |
| Meteor Korai | Pandy 38 | Nagy Angol |
| Montmorency | - | - |
| Nana | Crisana | - |
| Northstar | English Morello | Serbian Pie |
| Pamjat Vavilova | - | - |
| Pandy 38 | Landrace B | - |
| Pitic de Iasi | - | - |
| Rheinische Schattenmorelle | Landrace A | - |
| Schattenmorelle | Landrace A | - |
| Surefire | Borchert Black Sour | New York 6935 |
| Tamaris | - | - |
| Újfehértói Fürtös^2^ | - | - |
| Family A (n=67) | 25 14 (20) | 25 02 (29) |
| Family B (n=81) | M172 | 25 02 (29) |
| Family C (n=42) | Montmorency | 25 02 (29) |
| Family D (n=24) | Rheinische Schattenmorelle | Englaise Timpurii |
| Family E (n=78) | Újfehértói Fürtös | Surefire |

^1^Designates unknown parent

^2^Mutant of Pandy
